# Clinical and Laboratory Characteristics, Disease Activity, and Disease Damage: A Saudi National Lupus Cohort Study

**DOI:** 10.3390/healthcare14213775

**Published:** 2026-11-04

**Authors:** Ibrahim Almaghlouth, Najma Khalil, Haya M. Almalag, Lena M. Hassen, Kazi Nur Asfina, Hebatallah Hamed Ali, Eman Alqurtas, Mohamed Bedaiwi, Aos Aboabat, Jiandong Su, Fehaid G. Alanazi, Mohammed A. Omair, Abdurhman S. Alarfaj

**Affiliations:** 1 Rheumatology Unit, Department of Medicine, College of Medicine, King Saud University, Riyadh 11461, Saudi Arabia; ialmaghlouth@ksu.edu.sa (I.A.);; 2 College of Medicine Research Center, College of Medicine, King Saud University, Riyadh 11461, Saudi Arabia; 3 Department of Clinical Pharmacy, College of Pharmacy, King Saud University, Riyadh 11451, Saudi Arabia; 4 Department of Zoology, College of Sciences, King Saud University, Riyadh 11451, Saudi Arabia; 5 University Health Network, University of Toronto, Toronto, ON M5T 2S8, Canada

**Keywords:** systemic lupus erythematosus, SLEDAI-2K, SLICC/ACR damage index, SDI, disease activity, disease damage

## Abstract

**Background**: Systemic lupus erythematosus (SLE) disease activity and damage assessments are crucial for understanding the disease burden across different lupus populations. This study aims to describe the disease characteristics, treatment, and disease activity and damage in the Saudi national prospective SLE cohort. **Methods**: Saudi SLE patients over 18 years of age were recruited prospectively from January 2020 to December 2023 from the national prospective cohort of SLE, Saudi Arabia. Data retrieved included patients’ sociodemographic characteristics, medical history, clinical manifestations and laboratory features, comorbidities, and medications at baseline visit, first follow-up at 3 months, and subsequent follow-up visits every 6 months. The SLE disease activity index 2000 (SLEDAI-2K) and Systemic Lupus International Collaboration Clinics/American College of Rheumatology (SLICC/ACR) damage index (SDI) scores were computed at baseline and follow-up visits following a standardized protocol. The generalized linear model was employed to assess organ damage based on the SDI scores. Kaplan–Meier survival analysis was performed to assess major adverse cardiovascular events (MACEs) and mortality. The Cox regression model was used to adjust for lupus disease and cardiovascular risk factors. **Results**: The mean age at SLE diagnosis of the 488 patients was 30.6 ± 11.9 years. The mean SLEDAI-2K and SDI scores were 8.0 ± 7.2 and 0.61 ± 1.1, respectively. The SLEDAI-2K scores declined initially and increased in follow-up visits. The SDI scores also increased at the follow-up visits. Musculoskeletal organ damage was found in 10.7% of the patients, neuropsychiatric damage in 9.2%, skin damage in 6.1%, renal damage in 5.9%, and cardiovascular damage in 4.4%. The parameters analyzed for predicting organ damage progression did not reach statistical significance, which may be due to the limited statistical power of the study and may not reflect an absence of relationship. Oral prednisolone was administered to 43.1% of the patients and hydroxychloroquine to 89.2%. The Kaplan–Meier 5- and 10-year survival rates were 99% and 98%, respectively. The initial findings using Cox regression analysis indicated that a longer SLE duration is protective of MACEs. However, a sensitivity analysis with a time-dependent approach did not retain significance of the SLE duration on the outcome of early MACEs. There was a trend of obesity and earlier MACE occurrence. **Conclusions**: Lupus disease activity and disease damage increased from the baseline visit over time. The most prevalent organ damage included musculoskeletal, neuropsychiatric, renal, and cardiovascular systems, consistent with previous studies. Time-invariant analysis showed an association between a longer SLE duration and MACEs, but such findings did not retain significance with a time-dependent approach, which may be due to immortal time bias. Obesity was found to contribute to early MACEs.

## 1. Introduction

Systemic lupus erythematosus (SLE) is a chronic multisystem heterogeneous autoimmune disease that manifests with various phenotypes and disease outcomes attributed to diverse ethnic and environmental factors [1,2]. The management and survival rates of SLE patients have improved markedly in recent decades. However, the morbidity rate due to the course of this disease and its therapy, which cause irreversible organ damage, remains high [3]. Irreversible organ damage is accrued during SLE, increasing the risk of death [4,5]. The assessment of the disease activity and organ damage are crucial for preventing damage accrual and predicting mortality [6,7].

Hopkins’ lupus cohort showed that older age at diagnosis, ethnicity, and low income were predictors of the advancement of disease damage [8]. Some studies showed that African-Americans suffered more damage than Caucasians, while others did not observe this [9,10]. Corticosteroid use was found to be strongly correlated with damage accrual, while hydroxychloroquine (HCQ) was found to have a protective effect on organ damage in SLE patients [11,12]. These cohorts serve as exemplary models and form the cornerstone of many SLE studies. They enable a deeper understanding of disease behavior by facilitating research into clinical manifestations, organ-specific outcomes, prognosis, and risk factors. Furthermore, they support vital investigations into clinical–laboratory correlations, as well as therapeutic efficacy and tolerability.

Despite clear evidence that ethnicity dictates SLE outcomes, there is a lack of data for Middle Eastern and Arab demographics, which forces clinicians to rely on extrapolated data from international cohorts that may not accurately represent these populations. To address this gap, establishing localized longitudinal cohort studies dedicated to these specific ethnicities is essential. Furthermore, the clinical profile of SLE in Saudi Arabia remains poorly defined, particularly within a unique genetic landscape marked by high consanguinity. Hence, there is a strong need to conduct a prospective study of Saudi SLE patients. The Saudi population with SLE has not been well characterized; although a few studies have described the associated clinical phenotypes via retrospective chart review and cross-sectional studies, they may be subject to several inherent biases [13,14]. To avoid these biases, we launched a prospective open design cohort study of SLE in January 2020 to study the nature of this disease in the Saudi population and assess the long-term risk of damage [15].

In this study, which is part of our national SLE (NSLE) cohort prospective study (protocol published in 2021), we aim to describe the clinical and laboratory characteristics, therapy, comorbidities, and course of disease activity, as well as to assess the disease activity and disease damage, to describe the cohort behavior over time and highlight the disease burden in the patients enrolled in the national prospective cohort of SLE, Saudi Arabia [15].

## 2. Materials and Methods

### 2.1. Study Design

This national prospective cohort study was initiated in January 2020, and patients were recruited until December 2023.

### 2.2. Patients and Eligibility Criteria

Saudi SLE patients over 18 years of age were enrolled from the Specialized Rheumatology Clinics at King Khalid University Hospital, Riyadh, after obtaining informed consent; these patients fulfilled the classification criteria for an SLE diagnosis of the American College of Rheumatology (ACR), Systemic Lupus International Collaboration Clinics (SLICC), or ACR/European League Against Rheumatism [16,17,18]. The study was approved by the institution’s ethics committee (Institutional Review Board No. E-19-3955/58; 15 December 2019) and was carried out in accordance with the principles of the Declaration of Helsinki.

### 2.3. Exclusion Criteria

Patients who did not fulfil the above criteria of eligibility, which included nationality, age, and disease diagnosis, were excluded.

### 2.4. Exposure and Outcome

We recorded data pertaining to the patients’ sociodemographic characteristics, medical history, clinical manifestations, laboratory features, comorbidities, and medications at the baseline visit, the first follow-up at 3 months, and subsequent follow-up visits every 6 months. Exposures included age, gender, SLE duration, and medications, and traditional CVD risk factors included smoking and comorbidities such as hypertension, diabetes, dyslipidemia, and obesity. Comorbidities (preexisting and newly diagnosed) and medications were recorded at the baseline and follow-up visits. Hypertension is defined as a blood pressure of ≥140/90 mm Hg by conventional definition, diabetes is defined as HbA1C > 5.7% or a fasting blood sugar > 100 mg/dL, liver disease or dyslipidemia is defined as a total cholesterol level of >5.2 or HDL < 0.77 or LDL > 3.4 mmol/L, obesity is defined as a BMI ≥ 25, weight loss is defined as an unintentional loss of 5% body weight over 6–12 months, and cancer was diagnosed by the oncology clinic. The disease activity in SLE patients was measured using the SLE disease activity index 2000 (SLEDAI-2K), and disease-related organ damage was measured over this period using the SLICC/ACR damage index (SDI) [15,16,17]. All variables related to the computation of the SLEDAI-2K and SDI scores were noted. We examined the changing trends in SLE disease burden regarding long-term outcomes (disease activity, disease damage, and mortality).

### 2.5. Statistical Analysis

Descriptive statistics were presented as the mean and standard deviation, and categorical variables were described as counts (percentages). Longitudinal measurements such as SLEDAI-2K and SDI were plotted as trend lines in terms of the mean values over repeated instances. The time-dependent generalized linear model of regression with Poisson distribution was employed to assess the outcome of the SDI score increase over the follow-up visits, to identify the predictors of organ damage progression, and overdispersion was corrected using Pearson scaling. Kaplan–Meier survival curves of major adverse cardiovascular events (MACEs) were plotted: a MACE was defined as any history of myocardial infarction (MI), stroke, venous thromboembolism (VTE), or cardiovascular disease (CVD)-related death, and it was presented as the number of patients at risk by years and the median survival year (95% CI). Time zero was considered the SLE diagnosis date. The event date was the earliest date of any outcome mentioned in the MACE definition. Censoring was defined for non-MACE patients when they lost follow-up or reached the last follow-up visit. The multivariable Cox regression model was used to adjust for SLE disease-specific and cardiovascular risk factors, the step-down variable selection method was used, and the Akaike information criterion (AIC) was adopted for the model fitting statistics. The proportional hazards assumption was checked using martingale residuals with 1000 replications. We also implemented a time-independent Cox model using patients’ enrollment date as time zero; time-varying covariates were used in the regression. Patients’ median follow-up time was calculated using the inverse Kaplan–Meier method. The software package SAS v.9.4 (SAS Institute Inc., Cary, NC, USA) was used to perform these analyses.

## 3. Results

**Figure 1:**
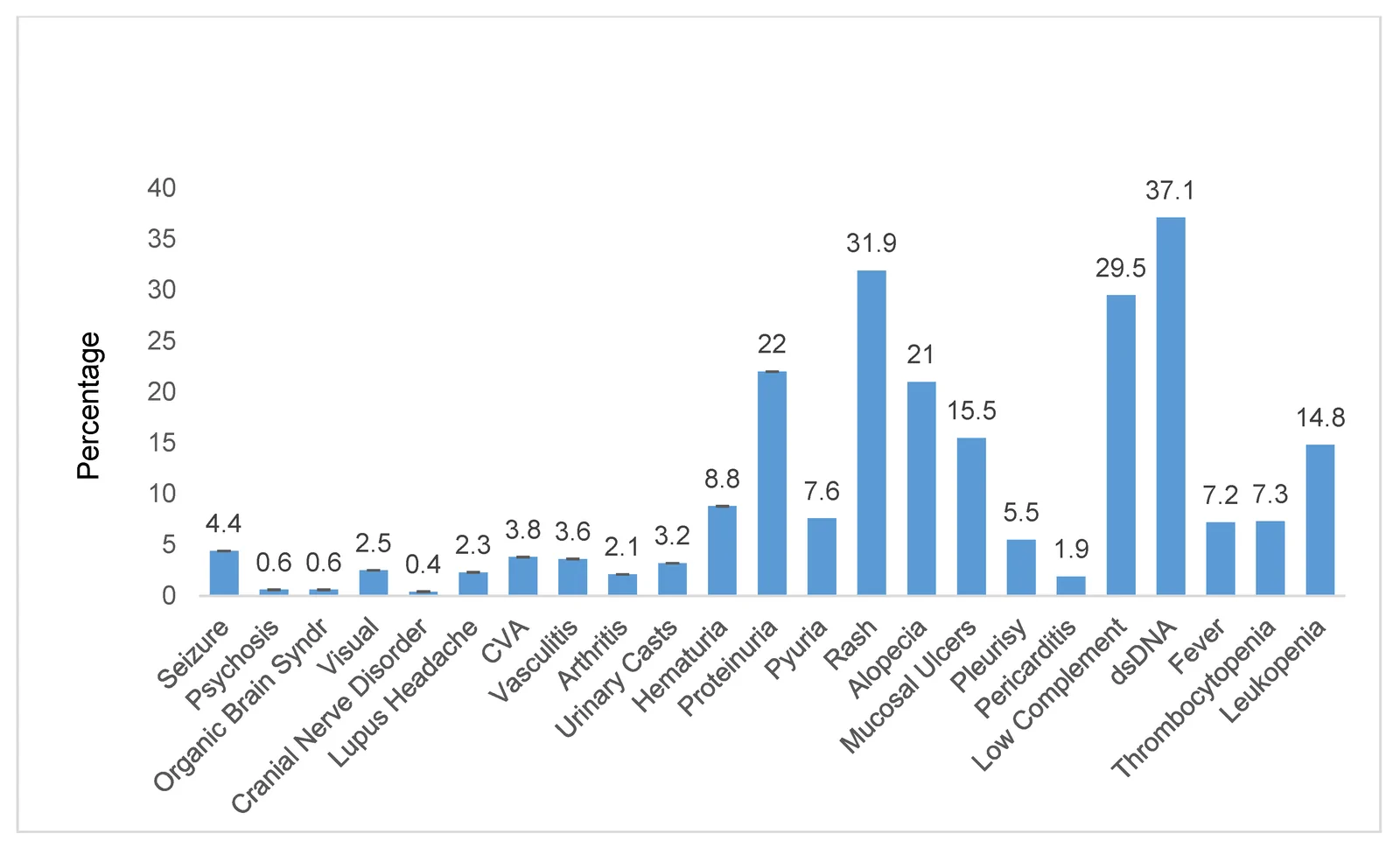
Baseline SLEDAI-2K graph representing the frequencies of clinical and laboratory characteristics in the lupus cohort.

**Figure 2:**
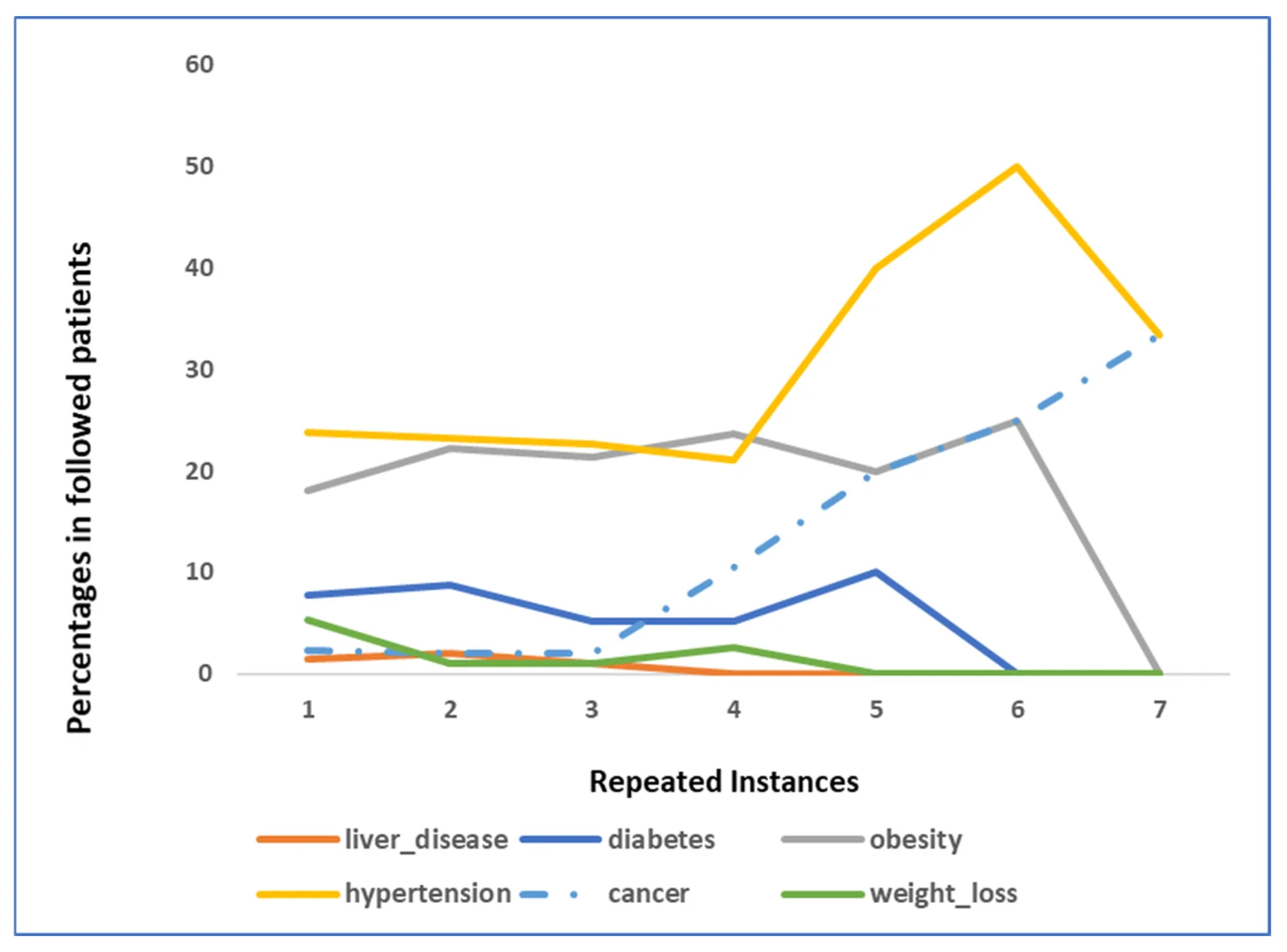
The trend of comorbidities at baseline and follow-up visits in the lupus cohort.

This prospective longitudinal study included a total of 488 SLE patients. The median of the follow-up years was 10.0 with an inter-quartile range of 5.2 to 16.1 years. The number of clinic visits with the first follow-up at 3 months and visits every 6 months thereafter ranged from one to nine, resulting in 985 visits. The baseline characteristics of this lupus cohort are summarized in Table 1. The mean age at SLE diagnosis was 30.6 ± 11.9 years, and the mean SLE disease duration was 10 ± 7.7 years. Of the 488 patients, 442 (90.6%) were female, and 46 were male (9.4%); the female-to-male ratio was 9.6:1. Seventy-eight patients (28.9%) had dyslipidemia, and their mean total cholesterol was 4.7 ± 1.2 mmol/L. Obesity (BMI ≥ 25) was observed in 63% of the patients and diabetes in 7.8%. Approximately, 96.9% of the patients tested positive for antinuclear antibodies (ANA), and 85.4% tested positive for anti-dsDNA antibodies. Lupus nephritis was found in 189 out of the 456 (41.4%) biopsied patients. The frequencies of the baseline clinical and laboratory characteristics based on the SLEDAI-2K scores are illustrated in Figure 1. Comorbidities including liver disease, diabetes, obesity, hypertension (HTN), dyslipidemia, cancer, and weight loss were observed over repeated visits. The trend of most comorbidities intensified with time, whereas the frequencies of liver disease and weight loss decreased (Figure 2).

**Figure 3:**
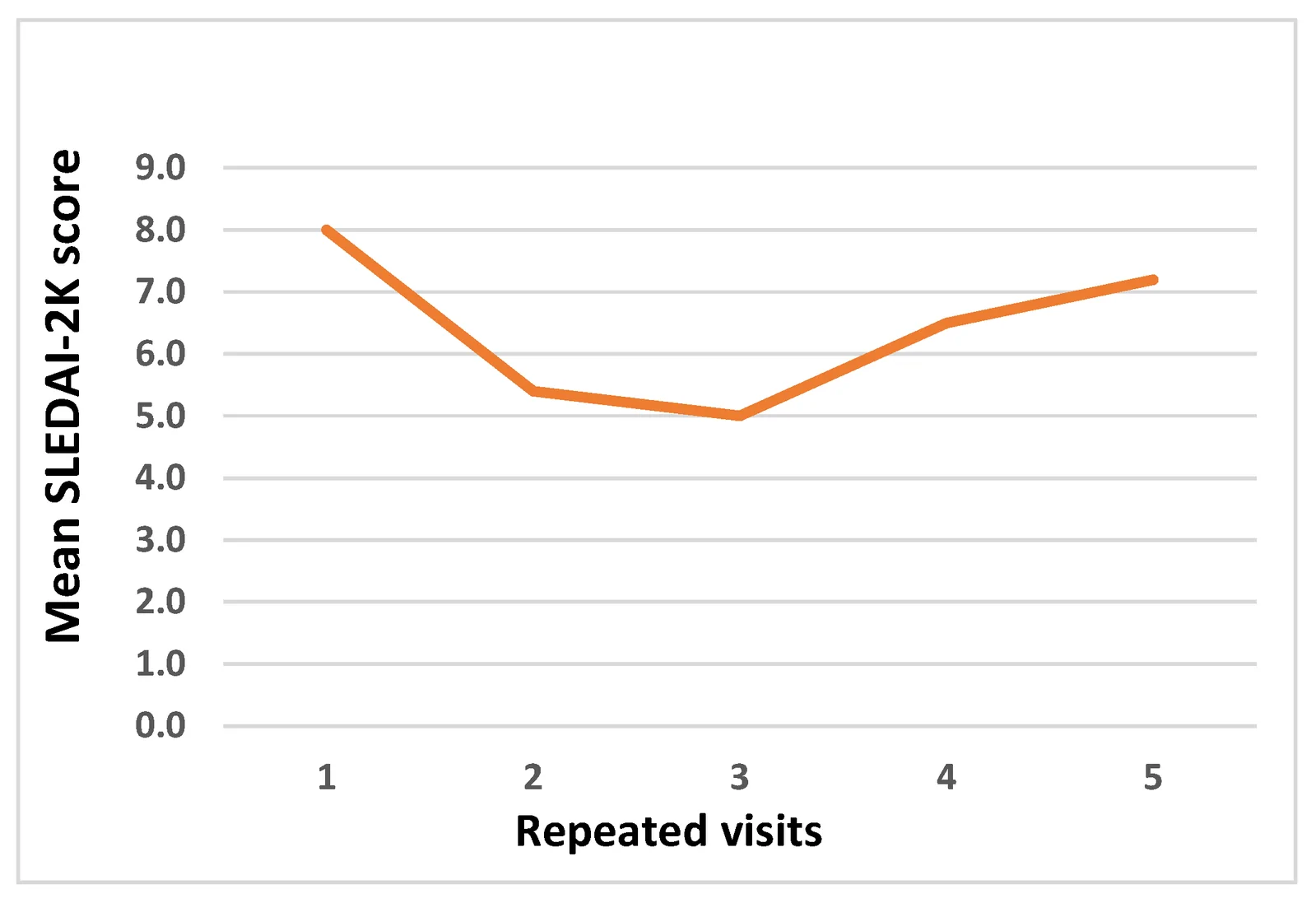
Mean SLEDAI-2K scores at baseline and follow-up visits in the lupus cohort.

The mean SLEDAI-2K score was 8.0 ± 7.2, and the mean SDI score was 0.6 ± 1.1. The SLEDAI-2K scores of the 488 patients over repeated visits are presented in Figure 3. The mean SLEDAI-2K score at the baseline visit was 8.0 ± 7.2, that at the second visit was 5.4 ± 6.7, that at the third visit was 5.0 ± 6.1, that at the fourth visit was 6.5 ± 8.0, and that at the fifth visit was 7.2 ± 8.6 (3 months, 9 months, 15 months, and 21 months after the baseline visit). The SLEDAI-2K scores decreased initially over the first three follow-up visits and then increased slightly; thereafter, they remained stable.

**Figure 4:**
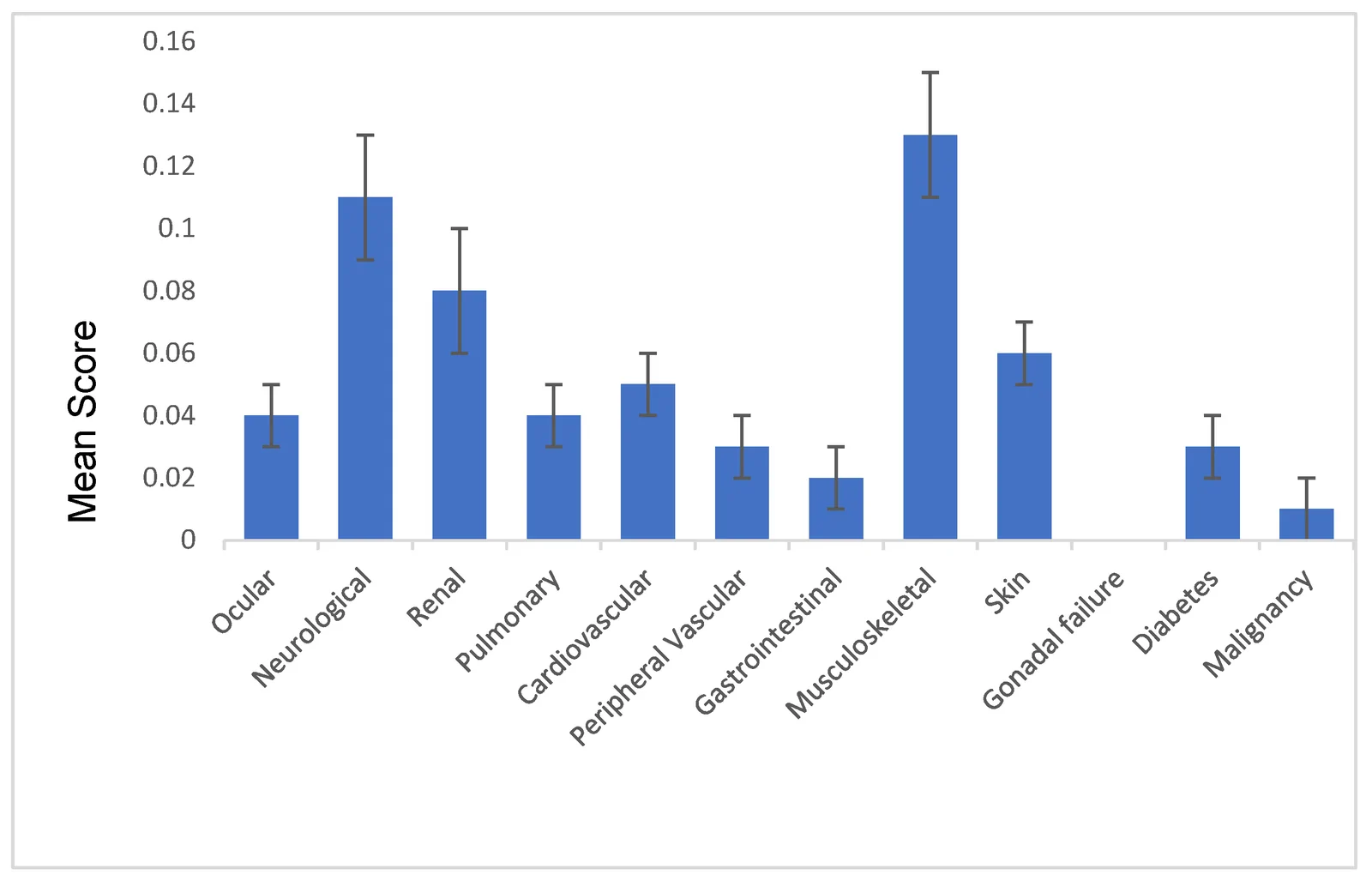
Baseline SDI organ system mean scores with standard error bars in the lupus cohort.

The baseline SDI organ system mean scores with standard error bars are presented in Figure 4. The SDI scores of different organ systems in the baseline visit and the follow-up visits are listed in Table 2. These SDI scores increased from the baseline visit (0.61 ± 1.05) to the fourth visit (0.84 ± 1.11) (15 months after the baseline visit). Organ damage (SDI score ≥ 1) was more prevalent in the musculoskeletal system (10.7%), followed by the neuropsychiatric system (9.2%), skin (6.1%), renal system (5.9%), and cardiovascular system (4.4%) compared to that in the other systems (Table 3). The number of patients who had an increased SDI score was limited; only 34 of the 985 visits (3.5%) (10 missing data) had a visit with >1 SDI score increase. The regressions were under-powered, and none of the predictors of organ damage progression reached statistical significance using all visit data according to the generalized linear model with Poisson distribution. This method considered intra-patient variations within same patient on repeated visits. The model was corrected for overdispersion using Pearson scaling (Table 4).

Regarding treatment, 43.1% of the lupus patients received the corticosteroid oral prednisolone (PSL), 89.2% received HCQ, and 43.5% were treated with different immunosuppressant drugs, including mycophenolate mofetil (MMF), azathioprine (AZA), and methotrexate (MTX). The percentage of patients on MMF therapy decreased over the follow-up visits (from 21.7% in the baseline visit to 14.3% in the follow-up visits), HCQ therapy ranged between 85.7% and 89.2% over the follow-up visits, AZA therapy (15%) remained constant, while MTX therapy (7.5% in the baseline visit, 14.3% in the fifth visit) was prescribed more frequently in the subsequent visits. The percentage of patients on belimumab declined over time, while those on rituximab increased from 2.1% at the baseline visit to 14.3% at the fourth visit. In these treatments, antihypertensives, statins, anticoagulants, vitamin D, and calcium carbonate were prescribed as well.

**Figure 5:**
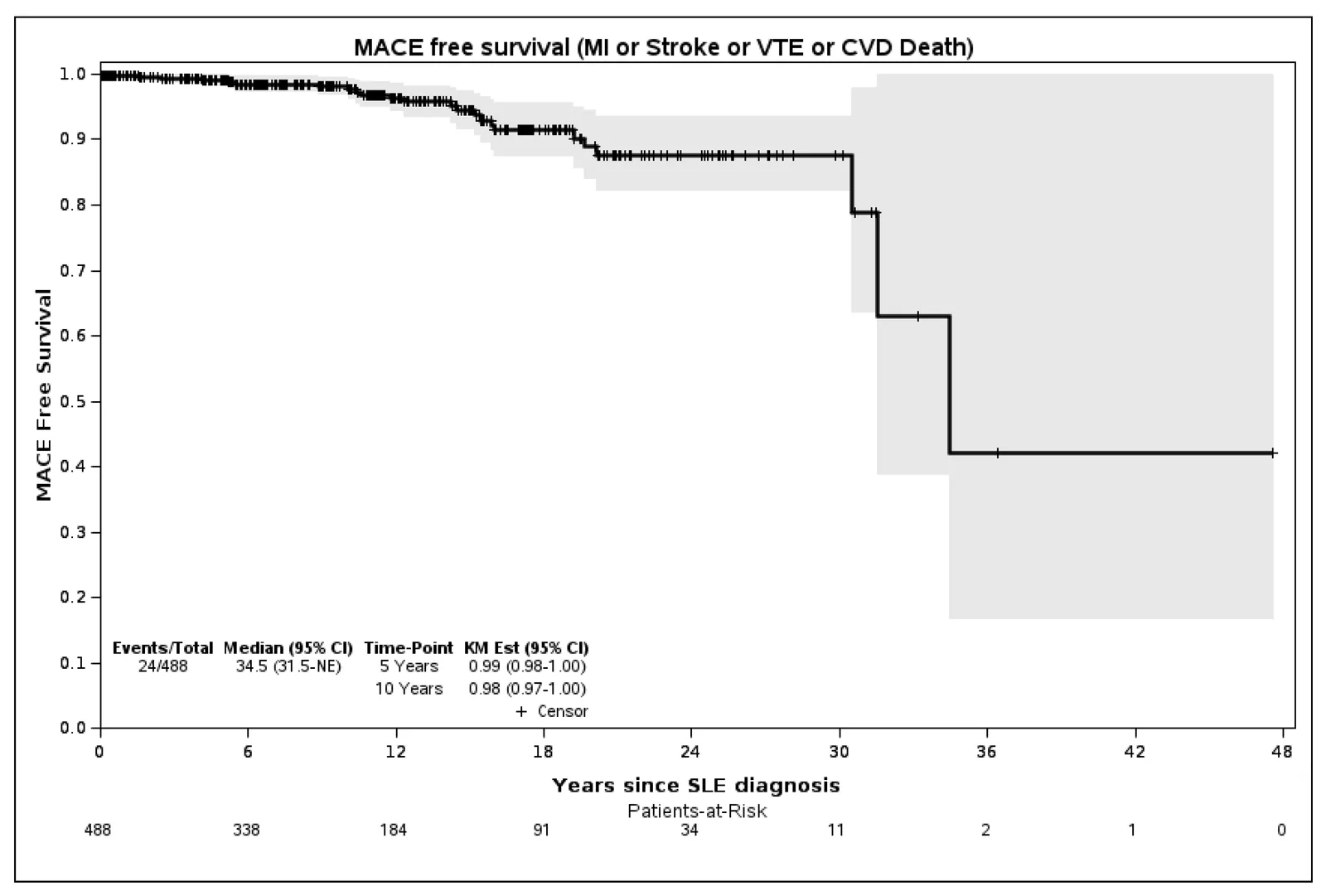
Kaplan–Meier survival curve of the lupus cohort with number of patients at risk by years and the median survival year (95% CI); 5-year survival 99% and 10-year survival 98%. MACE: major adverse cardiovascular events; MI: myocardial infarction; VTE: venous thromboembolism; CVD, cardiovascular disease-related death.

According to the Kaplan–Meier survival curve plotted in terms of the time to MACE, 24 events occurred during 48 years’ worth of follow-ups in the 488 patients. The median event-free survival duration was 34.5 years; the lower 95% CI was 31.5 years, and the upper 95% CI was not reached. The 5- and 10-year survival rates were 99% and 98%, respectively (Figure 5). In the time-invariant multivariable Cox proportional hazards regression analysis, a longer SLE disease duration was independently associated with a statistically significant reduction in the hazard of major adverse cardiovascular events (MACEs), after adjustment for potential confounders. Patients treated with statin for dyslipidemia had a statistically higher risk of MACE after adjusting for other covariates (Table 5). The residual-based proportional hazards assumption tests/plots indicated no violations of covariates’ proportionality of risk over time.

The sensitivity analysis did not show statistical significance of the SLE duration and statin treatment on the outcome of early MACEs. The model used the baseline age and gender, and the remaining covariates (classical cardiovascular risk factors (Table 6), SLE disease activity, treatment and lupus nephritis) were time-varying covariates. There was a trend of obesity (BMI ≥ 25) of the earlier MACE occurring with a Hazard Ratio (95% CI) of 6.15 (0.77, 49.06) and a *p* value of 0.087.

## 4. Discussion

Our national prospective lupus cohort study was conducted to address deficiencies in the existing knowledge of SLE disease in Saudi patients, because the current literature on SLE in Saudi populations is focused on retrospective chart reviews, which may be affected by biases [13,14]. Our study is the first prospective lupus cohort study among Saudi patients that adds to the knowledge on variations in SLE disease manifestations, disease activity, and disease damage in this ethnicity. The mean age at disease onset/diagnosis has been reported as 25–30 years in various studies worldwide [19,20,21,22]. The mean age reported in our study (30.6 ± 11.9 years) is consistent with these results, but it is higher than that reported in a previous retrospective study in our region (25.3 ± 11.4 years) [13]. The female-to-male ratio of 9.6:1 is similar to the ratio reported elsewhere, as well as in the previous study from our region [13,19,21,22]. A few studies have reported higher numbers of males and female-to-male ratios of 4.6:1 to 6.6:1 [23,24,25]. The incidence of lupus nephritis was lower in our cohort than that in a previous study from our region (41.4% vs. 47.9%) [13].

The presence of comorbid conditions increases the disease damage in SLE. Hypertension is present as a comorbidity in approximately one-third of all SLE patients, and it increases the frequency of cardiovascular events and the severity of renal damage [8,26,27]. HTN was less prevalent in our cohort compared with that in previous studies [21,26].

The mean baseline SLEDAI-2K scores reported in the literature range from 5.3 to 6.9 [4,22,23,28]. The mean baseline SLEDAI-2K score in our study was higher at 8.0 ± 7.2. However, it decreased to 5.4 ± 6.7 at the first follow-up visit. In general, the disease activity based on SLEDAI-2K decreases over time and remains lower in the first 5 years [23]. In our study, it decreased at first, increased slightly thereafter, and stabilized eventually. Ethnicity influences the SLEDAI-2K score/lupus disease activity in lupus patients [23]. A more severe disease activity based on the mean SLEDAI-2K score of 12.2 was reported in the European inception cohort [29]. White lupus patients exhibited lower disease activity compared to non-Whites in the 5-year follow-up, demonstrating the effect of genetic factors on lupus. Patients of African origin had higher lupus disease activity levels than those of Whites. In the LUMINA cohort, Hispanics exhibited the highest disease activity, followed by African-Americans, whereas Whites exhibited the lowest disease activity among the different races [30].

In our SLE cohort, the mean SDI scores increased from 0.61 to 0.84 in subsequent visits, similar to the trend observed in a few previous studies, wherein the mean SDI score increased from 0.4 to 1.1 over 10 years [22]. In the present study, none of the parameters significantly predicted organ damage progression via regression analysis of the SDI scores. A comparison of the organ damage data obtained in our study with those reported in a few Asian studies is presented in Table 3 [19,20,21,22]. The SDI organ damage data obtained from these regions indicated that the musculoskeletal system had the most organ damage (6.4–32.1%), which is similar to our finding (10.7%) [20,22]. Other studies have reported renal (3.6–34.7%) and neuropsychiatric (8.8–22.3%) damage as the most common types of organ damage [21]. In our study, neuropsychiatric damage was the second most-frequent type of disease damage, followed by skin, renal, and cardiovascular organ damage; this result is similar to the findings in Asian cohorts. Overall, the disease burden of our cohort was lower than those of other cohorts in most damage domains, with a relatively higher burden of cardiovascular damage. In our recent study, we reported that although our SLE patients were younger and had fewer cardiovascular risk factors than the general population, they were at a higher risk of developing MACE [31]. Most of the risk of MACE development in our lupus cohort can seemingly be attributed to the disease instead of traditional cardiovascular risk factors, such as dyslipidemia, HTN, or diabetes [31,32]. Early diagnosis might be an important strategy, at least in the Saudi population, to mitigate several of the cardiovascular manifestations associated with the early course of the disease and subsequent accrued damage.

Several studies have reported the use of corticosteroids in almost 69% of SLE patients, and corticosteroid use stood at 96% in a study conducted in our region [4,13]. In our study, oral PSL was administered to 43.1% of the patients. Corticosteroid use is associated with an increase in disease damage [21,33]. Urowitz et al. suggested that SLE disease activity decreases in the first 5 years, and damage accumulates, particularly corticosteroid-therapy-related damage [23]. According to studies that have reported on SLE therapy in patients since 2000, the disease damage due to corticosteroids has not increased, unlike the case of patients treated before 2000, who were mostly treated with corticosteroids [23]. We found a decline in the use of corticosteroids over the last decade in our center compared with that in a previous study from our region [13]. HCQ was administered to 89.2% of the patients in our cohort compared to 67% in a previous study conducted in our region [13]. AZA use has declined from 32% in our region to 15% in our cohort, and MMF therapy has increased from 8.2% to 21.7% [13]. Regarding therapy, the LUMINA and Toronto groups have proposed that HCQ use is associated with reduced organ damage [34,35]. In our study, 89.2% of the patients were administered HCQ, which is similar to the 86–95.7% HCQ use rate reported in studies supporting HCQ use to protect against organ damage in SLE patients [20,21]. Persistent disease activity increases damage accrual, as reported in previous studies, and some studies have advocated the need for additional therapies to optimally control disease activity, which is often not achieved with the available steroid-sparing treatment options [29,36,37].

In a meta-analysis published by Mak et al. in 2012, the global trend of SLE from the 1990s to the 2000s in terms of the pooled 5-year survival rate was 92.7–94.8% and that in terms of the pooled 10-year survival rate was 88.1–91.4% [38]. Additionally, they reported that the 5- and 10-year survival rates of Asians (88% and 84%, respectively) were lower than those of Europeans (93% and 91%, respectively). Another study categorized the SLE patients diagnosed between 1995 and 2004 and those diagnosed between 2005 and 2018 into two groups [39]. The 5- and 10-year cumulative survival rates of the first group diagnosed in the earlier years were 93.6 and 91.0%, respectively, and those of the second group diagnosed in the later years were 96.5 and 94.2%. This improvement in SLE prognosis over the years was mainly attributed to a substantial reduction in organ damage accrual, associated with the cautious use of glucocorticoids and early use of glucocorticoid-sparing agents in the current era. The 5- and 10-year survival rates computed using the Kaplan–Meier survival curve in our study are comparable with those reported in other studies conducted elsewhere, as well as with those reported in the studies conducted in our region, with a trajectory of continuous improvement [13,40]. The time-invariant multivariable Cox model showed that a longer SLE diagnosis is associated with a lower risk for MACE. While this might be attributed to lupus related thrombosis rather than atherosclerosis, cautious interpretation is warranted given the small number of events and the risk of immortal time bias. To minimize the risk of immortal time bias we conducted a time-dependent Cox regression model, which, in turn, did not show an association between SLE duration and statin use. In addition, obesity may impact earlier MACEs in lupus patients. However, this should be interpreted with caution knowing the trend nature of the association related to small number of events.

### Limitations

This is a single-center observational study with the constraints of referral bias, immortal time bias, missing data, and a small number of events; hence, there should be cautious interpretation, and generalizability may be affected. However, its prospective nature allows us to assess disease characteristics over time. There were several patients with a long duration between their SLE diagnosis and study enrollment; the disease-specific and treatment information during this period might be missing or may have introduced left censoring bias for those who did not make the enrollment date. We performed a sensitivity analysis using enrollment data to try to remediate this shortcoming. Second, the low number of events of SDI increase and MACE limited the power of statistical analysis; even though we used longitudinal data with repeated visits for each patient, the event-to-covariate ratio was still under 10 events per covariate.

The primary goal was to explore the nature and pattern of disease phenotypes and disease burden. Further analysis may be needed to explore other contributing factors.

This study represents an important foundation for the proper assessment of the current picture of lupus disease in the Saudi population. To overcome the limitation of generalizability, we intend to collaborate with other national and international centers. By using an open cohort design, we minimized many of the inherent biases affecting observational studies, but some biases remained. Our study design allowed people to enroll at any time in their disease course and leave freely. To minimize the risk of withdrawal from study, we engaged all patients with whom contact was lost for more than 6 months (or those that deviated from the original study protocol) through follow-up phone calls to identify the reasons why they terminated their enrollment. Furthermore, we focused on the accumulated burden and prevalence of comorbidities rather than the initial manifestations. Given the descriptive nature of our analysis, the findings of this study should be useful for generating hypotheses and as areas of focus for our future research.

## 5. Conclusions

This is the first report of Saudi lupus phenotypes and the burden of the disease over time using a prospective cohort design. The clinical and laboratory characteristics of lupus disease observed are similar to previous reports elsewhere, including an earlier report from our region with a trajectory of continuous improvement. SLE disease activity and disease damage assessed by the SLEDAI-2K and SDI scores, respectively, show that the SLEDAI-2K decreased initially over the first three follow-up visits and then increased, while the SDI increased from the baseline visit over the next follow-up visits. Organ damage (SDI score ≥ 1) was more prevalent in the musculoskeletal system, followed by the neuropsychiatric, skin, renal and cardiovascular systems, with increased severity over time, similar to previous reports. None of the parameters analyzed to predict organ damage progression reached statistical significance via multivariate analysis of the SDI scores, which may be due to the limited statistical power of the study and may not reflect an absence of relationship. The findings of this study, including the relatively high survival rates observed in this population, warrant further investigation to identify potential contributing factors, particularly socioeconomic determinants of health, including access to medications, healthcare availability, and overall healthcare accessibility.

## Figures and Tables

**Table 1:** Baseline characteristics of the lupus cohort of 488 patients.

Characteristics	*n* (%)/Mean ± SD
Age (years)	
At enrolment	40.7 ± 12.5
At SLE diagnosis	30.6 ± 11.9
Gender	
Female	442 (90.6)
Male	46 (9.4)
Female:male	9.6:1
Smoker (*n* = 207)	7 (3.4)
SLE duration (years)	10.0 ± 7.7
Total cholesterol (mmol/L) (*n* = 270)	4.7 ± 1.2
HDL (mmol/L)	1.6 ± 0.5
LDL (mmol/L)	2.6 ± 0.9
Dyslipidemia (*n* = 270) (Total cholesterol > 5.2 or HDL < 0.77 or LDL > 3.4)	78 (28.9%)
Hypertension (*n* = 483) *By conventional hypertension definition*	
<140/90 mmHg	390 (80.7%)
≥140/90 mmHg	93 (19.3%)
*By American College of Cardiology/American Heart* *Association (ACC/AHA) hypertension definition*	
<130/80 mmHg	256 (53.0%)
≥130/80 mmHg	134 (27.7%)
≥140/90 mmHg	93 (19.3%)
Obesity (BMI ≥ 25) (*n* = 465)	293 (63.0%)
Diabetes (*n* = 460)	36 (7.8%)
Antinuclear antibody-positive (*n* = 254)	246 (96.9%)
Anti-dsDNA antibody-positive (*n* = 473)	404 (85.4%)
SLEDAI -2K Score	8.0 ± 7.2
SDI Score	0.6 ± 1.1
*Treatment (n = 480)*	
Oral prednisolone	207 (43.1)
Hydroxychloroquine	428 (89.2)
Immunosuppressants	209 (43.5)

*n* = 488 where not indicated.

**Table 2:** SDI scores of different organ systems at baseline and follow-up visits in the lupus cohort.

SDI Scores				
Organ System	Baseline Visit	Follow-Up	Follow-Up	Follow-Up
		3 m After Baseline Visit	9 m After Baseline Visit	15 m After Baseline Visit
	Mean ± SD	Mean ± SD	Mean ± SD	Mean ± SD
	First Visit	Second Visit	Third Visit	Fourth Visit
Ocular	0.04 ± 0.22	0.04 ± 0.23	0.04 ± 0.23	0.07 ± 0.31
Neuropsychiatric	0.11 ± 0.39	0.17 ± 0.49	0.13 ± 0.37	0.16 ± 0.44
Renal	0.08 ± 0.34	0.08 ± 0.33	0.08 ± 0.36	0.06 ± 0.39
Pulmonary	0.04 ± 0.22	0.06 ± 0.26	0.07 ± 0.27	0.06 ± 0.30
Cardiovascular	0.05 ± 0.27	0.04 ± 0.26	0.05 ± 0.29	0.05 ± 0.21
Peripheral vascular	0.03 ± 0.17	0.02 ± 0.15	0.03 ± 0.16	0.02 ± 0.12
Gastrointestinal	0.02 ± 0.14	0.01 ± 0.09	0.01 ± 0.11	0.02 ± 0.12
Musculoskeletal	0.13 ± 0.39	0.18 ± 0.44	0.16 ± 0.40	0.17 ± 0.42
Skin	0.06 ± 0.25	0.05 ± 0.22	0.07 ± 0.25	0.08 ± 0.27
PGF	0	0	0	0
Diabetes	0.03 ± 0.18	0.04 ± 0.19	0.04 ± 0.19	0.03 ± 0.17
Malignancy	0.01 ± 0.14	0.03 ± 0.19	0.04 ± 0.23	0.05 ± 0.21
SDI score	0.61 ± 1.05	0.83 ± 1.29	0.84 ± 1.19	0.84 ± 1.11

PGF: premature gonadal failure.

**Table 3:** Organ damage in SLE patients compared in different studies.

Organ Damage: SDI Score ≥ 1 (%)					
Organ System	Present Study	Raman et al. [19]	Wang et al. [20]	Ghazali et al. [21]	Joo and Bae [22]
Year	2023	2020	2018	2018	2015
Ocular	4.2	10.3	9.5	5.3	8.9
Neuropsychiatric	9.2	22.0	17.9	22.3	8.8
Renal	5.9	34.7	3.6	4.3	11.1
Pulmonary	3.6	9.0	10.7	4.3	7.0
Cardiovascular	4.4	5.0	4.8	3.2	3.1
Peripheral vascular	2.3	5.3	9.5	0	2.7
Gastrointestinal	1.9	2.3	1.2	0	0.9
Musculoskeletal	10.7	20.3	32.1	6.4	14.9
Skin	6.1	-	-	12.8	3.7
Premature gonadal failure	0	4.7	8.3	6.4	0.7
Diabetes	3.3	6.0	2.4	0	2.6
Malignancy	1.3	6.3	-	0	3.9

**Table 4:** Outcome of SDI score increase over the follow-up visits according to the generalized linear model with Poisson distribution in the lupus cohort.

Parameters	Univariate Poisson Regression					Multivariable Poisson Regression			
	Relative Risk	Lower Confidence Limit of Relative Risk	Upper Confidence Limit of Relative Risk	Pr > Chi-Sq	Variable Selection	Relative Risk	Lower Confidence Limit of Relative Risk	Upper Confidence Limit of Relative Risk	Pr > Chi-Sq
Age at SLE diagnosis	1.00	0.96	1.04	0.947	Step down until smallest AIC reached				
Female vs. male	1.25	0.23	6.80	0.795					
SLE duration in years	1.00	0.94	1.06	0.920					
Conventional hypertension	1.82	0.72	4.64	0.209		1.65	0.66	4.15	0.286
Obesity (BMI ≥ 25)	0.63	0.27	1.49	0.295		0.64	0.27	1.49	0.298
Diabetes	0.49	0.05	5.06	0.547					
Statin treatment	0.84	0.08	8.78	0.881					
SLEDAI-2K score	1.00	0.94	1.07	0.958					
Treated with steroids	0.53	0.19	1.44	0.212		0.51	0.19	1.36	0.179
Treated with hydroxychloroquine	1.46	0.27	7.90	0.660					
Treated with immunosuppressants	0.93	0.38	2.26	0.866					
Biopsy-proven lupus nephritis	1.80	0.74	4.37	0.197		1.82	0.76	4.34	0.178

Except for the first two covariables of age and gender, all other covariates are time-dependent.

**Table 5:** Cox regression for the outcome of the cardiovascular risk factors, MACE (MI or stroke or VTE or CVD death), in the lupus cohort.

Baseline Variables	Univariate Cox Regression				Multivariable Cox Regression			
	Hazard Ratio	Lower Confidence Limit of Hazard Ratio	Upper Confidence Limit of Hazard Ratio	Pr > Chi-Sq	Hazard Ratio	Lower Confidence Limit of Hazard Ratio	Upper Confidence Limit of Hazard Ratio	Pr > Chi-Sq
Age at SLE diagnosis	1.04	1.00	1.08	0.083				
Female vs. male	0.74	0.21	2.59	0.642				
SLE duration in years	0.60	0.51	0.70	<0.0001	0.55	0.46	0.66	<0.0001
Conventional hypertension	1.03	0.37	2.88	0.950				
Obesity (BMI ≥ 25)	0.80	0.32	2.00	0.627				
Diabetes	0.71	0.16	3.08	0.645				
Statin Treatment	2.05	0.47	8.94	0.340	5.48	1.12	26.73	0.035
SLEDAI-2K score	1.04	0.99	1.10	0.131	1.05	0.99	1.11	0.112
Treated with steroids	1.10	0.46	2.66	0.825				
Treated with hydroxychloroquine	0.63	0.21	1.88	0.405				
Treated with immunosuppressives	1.12	0.46	2.76	0.800				
Biopsy-proven lupus nephritis	0.77	0.32	1.86	0.557				

MACE: major adverse cardiovascular events; MI: myocardial infarction; VTE: venous thromboembolism; CVD: cardiovascular disease-related death.

**Table 6:** Cox regression for the outcome of cardiovascular risk factors, MACE (MI or Stroke or VTE or CVD death), in the lupus cohort (time-dependent approach; note that the first two predictors are fixed at baseline, while the remaining variables are time-dependent).

Baseline Variables	Hazard Ratio	Lower Confidence Limit of Hazard Ratio	Upper Confidence Limit of Hazard Ratio	Pr > Chi-Sq	Variable Selection	Hazard Ratio	Lower Confidence Limit of Hazard Ratio	Upper Confidence Limit of Hazard Ratio	Pr > Chi-Sq
Age at SLE diagnosis	1.02	0.97	1.07	0.562	Step down until smallest AIC reached				
Female vs. male	0.38	0.08	1.80	0.222		0.40	0.09	1.92	0.254
SLE duration in years	0.98	0.89	1.07	0.581					
Conventional hypertension definition	1.73	0.45	6.67	0.430					
Obesity (BMI ≥ 25)	5.61	0.71	44.46	0.102		6.15	0.77	49.06	0.087
Diabetes	1.38	0.17	10.88	0.763					
Statin treatment	2.55	0.32	20.13	0.375		4.24	0.47	38.32	0.198
SLEDAI-2K score	1.03	0.96	1.11	0.417					
Treated with steroids	1.36	0.39	4.73	0.632					
Treated with antimalarials	0.46	0.10	2.18	0.330					
Treated with immunosuppressants	1.99	0.56	7.08	0.289		3.05	0.80	11.65	0.102
Biopsy-proven lupus nephritis	0.35	0.07	1.64	0.183		0.21	0.04	1.11	0.066

MACE: major adverse cardiovascular events; MI: myocardial infarction; VTE: venous thromboembolism; CVD: cardiovascular disease-related death.

## Data Availability

The data presented in this study are not publicly available due to privacy and ethical restrictions. Further inquiries can be directed to the corresponding author.

## Abbreviations

The following abbreviations are used in this manuscript:

SLE	Systemic lupus erythematosus
SLEDAI-2K	SLE disease activity index 2000
SLICC	Systemic Lupus International Collaborating Clinics
ACR	American College of Rheumatology
SDI	SLICC/ACR Damage Index
HCQ	Hydroxychloroquine
MACE	Major adverse cardiovascular events
MI	Myocardial infarction
VTE	Venous thromboembolism
CVD	Cardiovascular disease
CI	Confidence interval
BMI	Body mass index
ANA	Antinuclear antibodies
anti-dsDNA	Anti-double stranded deoxyribonucleic acid antibodies
HTN	Hypertension
PSL	Prednisolone
MMF	Mycophenolate mofetil
AZA	Azathioprine
MTX	Methotrexate
LUMINA	Lupus in minorities: nature vs. nurture

## References

[1] Barber M.R.W., Drenkard C., Falasinnu T., Hoi A., Mak A., Kow N.Y., Svenungsson E., Peterson J., Clarke A.E., Ramsey-Goldman R. (2021). Global epidemiology of systemic lupus erythematosus. Nat. Rev. Rheumatol..

[2] Lewis M.J., Jawad A.S. (2017). The effect of ethnicity and genetic ancestry on the epidemiology, clinical features and outcome of systemic lupus erythematosus. Rheumatology.

[3] Touma Z., Kayaniyil S., Parackal A., Bonilla D., Su J., Johnston A., Gahn J., Hille E.D., Ohsfeldt R., Chandran S. (2024). Modelling long-term outcomes for patients with systemic lupus erythematosus. Semin. Arthritis Rheum..

[4] Bruce I.N., O’Keeffe A.G., Farewell V., Borba E.F., Urowitz M.B., Gladman D.D., Peschken C., Bae S.C., Fortin P.R., Sanchez-Guerrero J. (2015). Factors associated with damage accrual in patients with systemic lupus erythematosus: Results from the Systemic Lupus International Collaborating Clinics (SLICC) Inception Cohort. Ann. Rheum. Dis..

[5] Pri-Paz Basson Y., Haim-Pinhas H., Erez D., Sagy I., Cohen-Hagai K., Kivity S., Tayer-Shifman O.E. (2026). Damage Accrual in Patients with Systemic Lupus Erythematosus Predicts Mortality and Is Associated Primarily with Antiphospholipid Syndrome and Hypertension. J. Clin. Med..

[6] Ramnarain A., Liam C., Milea D., Morand E., Kent J., Kandane-Rathnayake R. (2024). Predictors of organ damage in systemic lupus erythematosus in the Asia Pacific region: A systematic review. Arthritis Care Res..

[7] Hunnicutt J., Georgiou M.E., Richards A., Quasny H., Magder L., Goldman D., Petri M.A. (2024). Patients achieving low lupus disease activity state, systemic lupus erythematosus disease control or remission showed lower rates of organ damage during longitudinal follow-up: Analysis of the Hopkins Lupus Cohort. Lupus Sci. Med..

[8] Petri M., Purvey S., Fang H., Magder L.S. (2012). Predictors of organ damage in systemic lupus erythematosus: The Hopkins Lupus Cohort. Arthritis Rheumatol..

[9] Cooper G.S., Treadwell E.L., St Clair E.W., Parks C.G., Gilkeson G.S., Dooley M.A., Friedman J.Y., Joiner E.E., Kraft M., Rosenberg A.M. (2007). Sociodemographic associations with early disease damage in patients with systemic lupus erythematosus. Arthritis Rheumatol..

[10] Yee C.S., Hussein H., Skan J., Bowman S., Teh L.S., Gordon C. (2003). Association of damage with autoantibody profile, age, race, sex and disease duration in systemic lupus erythematosus. Rheumatology.

[11] Al Sawah S., Zhang X., Zhu B., Magder L.S., Foster S., Iikuni N., Petri M. (2015). Effect of corticosteroid use by dose on the risk of developing organ damage over time in systemic lupus erythematosus-the Hopkins Lupus Cohort. Lupus Sci. Med..

[12] Dima A., Jurcut C., Chasset F., Balanescu P., Badea C., Caraiola S., Cojocaru M., Bojinca V., Copotoiu M., Berghea F. (2022). Hydroxychloroquine in systemic lupus erythematosus: Overview of current knowledge. Ther. Adv. Musculoskeletal. Dis..

[13] Al Arfaj A.S., Khalil N. (2009). Clinical and immunological manifestations in 624 SLE patients in Saudi Arabia. Lupus.

[14] Abid N., Khan A.S., Al Otaibi F.H. (2013). Systemic lupus erythematosus (SLE) in the eastern region of Saudi Arabia. A comparative study. Lupus.

[15] Almaghlouth I.A., Hassen L.M., Alahmari H.S., Albalawi M.A., Al-Homood I.A., Alarfaj A.S., Omair M.A., Halabi H.E., Alosaimi H.M., Al-Rayes H.M. (2021). National systemic lupus erythematosus prospective cohort in Saudi Arabia: A study protocol. Medicine.

[16] Hochberg M.C. (1997). Updating the American College of Rheumatology revised criteria for the classification of systemic lupus erythematosus. Arthritis Rheumatol..

[17] Petri M., Orbai A.M., Alarcón G.S., Gordon C., Merrill J.T., Fortin P.R., Bruce I.N., Isenberg D., Wallace D.J., Nived O. (2012). Derivation and validation of the Systemic Lupus International Collaborating Clinics classification criteria for systemic lupus erythematosus. Arthritis Rheumatol..

[18] Aringer M., Costenbader K., Daikh D., Brinks R., Mosca M., Ramsey-Goldman R., Smolen J.S., Wofsy D., Alarcón G.S., Alexander R. (2019). 2019 European League Against Rheumatism/American College of Rheumatology Classification Criteria for Systemic Lupus Erythematosus. Arthritis Rheumatol..

[19] Raman L., Yahya F., Ng C.M., Sockalingam S.S. (2020). Early damage as measured by SLICC/ACR damage index is a predictor of hospitalization in systemic lupus erythematosus (SLE). Lupus.

[20] Wang Z., Li M., Wang Y., User P., Zhao J., Leng X., Tian X., Zhang X., Zeng X. (2018). Long-term mortality and morbidity of patients with systemic lupus erythematosus: A single-center cohort study in China. Lupus.

[21] Ghazali W.S.W., Daud S.M.M., Mohammad N., Awang M.S. (2018). SLICC damage index score in systemic lupus erythematosus patients and its associated factors. Medicine.

[22] Joo Y.B., Bae S.C. (2015). Assessment of clinical manifestations, disease activity and organ damage in 996 Korean patients with systemic lupus erythematosus: Comparison with other Asian populations. Int. J. Rheum. Dis..

[23] Urowitz M.B., Gladman D.D., Ibañez D., Fortin P.R., Bae S.C., Gordon C., Clarke A., Bernatsky S., Hanly J.G., Isenberg D. (2012). Evolution of disease burden over five years in a multicenter inception systemic lupus erythematosus cohort. Arthritis Care Res..

[24] Alarcón G.S., Friedman A.W., Straaton K.V., Moldovan J.N., Roseman J.M., Bastian H.M., McGwin G., Fessler B.J., Reveille J.D. (1999). Systemic lupus erythematosus in three ethnic groups: III. A comparison of characteristics early in the natural history of the LUMINA cohort. LUpus in MInority populations: NAture vs. Nurture. Lupus.

[25] Nazarinia M.A., Ghaffarpasand F., Shamsdin A., Moayedi J. (2008). Systemic lupus erythematosus in Fars province of Iran. Lupus.

[26] Wolfe F., Michaud K., Li T., Katz R.S. (2010). Chronic conditions and health problems in rheumatic diseases: Comparisons with rheumatoid arthritis, non-inflammatory rheumatic disorders, systemic lupus erythematosus, and fibromyalgia. J. Rheumatol..

[27] Mbengue M., Lot M.L., Diagne S., Niang A. (2021). Proliferative Glomerulonephritis: Risk Factor for Hypertension in Lupus. Int. J. Hypertens..

[28] Maloney K.C., Ferguson T.S., Stewart H.D., Myers A.A., De Ceulaer K. (2017). Clinical and immunological characteristics of 150 systemic lupus erythematosus patients in Jamaica: A comparative analysis. Lupus.

[29] Nossent J., Kiss E., Rozman B., Szekanecz Z., Cikes N., Varju C., Kovacs L., Zeher M., Houssiau F., Fritsch-Stork R. (2010). Disease activity and damage accrual during the early disease course in a multinational inception cohort of patients with systemic lupus erythematosus. Lupus.

[30] Zhang J., Gonzalez L.A., Roseman J.M., McGwin G., Albert D.A., Bastian H.M., Alarcón G.S., Vila L.M., Reveille J.D. (2010). Predictors of the rate of change in disease activity over time in LUMINA, a multiethnic US cohort of patients with systemic lupus erythematosus: LUMINA LXX. Lupus.

[31] Almaghlouth I., Bohuliga K.G., Alanazi B., Alrashid A., Hassen L.M., Alahmari H.S., Al-Homood I.A., Alarfaj A.S., Omair M.A., Halabi H.E. (2024). Prevalence of major adverse cardiovascular events among Saudi patients with systemic lupus erythematosus compared with the general population: Updates from the national SLE and PURE cohorts. Lupus Sci. Med..

[32] Md Yusof M.Y., Vital E.M. (2021). Early intervention in systemic lupus erythematosus: Time for action to improve outcomes and health-care utilization. Rheumatol. Adv. Pract..

[33] Kandane-Rathnayake R., Milea D., Louthrenoo W., Bae S.C., Li Z., Oon S., Golder V., Nikpour M., Harigai M., Zhang Z. (2025). Longitudinal associations of flare and damage accrual in patients with systemic lupus erythematosus. Lupus Sci. Med..

[34] Fessler B.J., Alarcón G.S., McGwin G., Roseman J.M., Bastian H.M., Friedman A.W., Reveille J.D. (2005). Systemic lupus erythematosus in three ethnic groups: XVI. Association of hydroxychloroquine use with reduced risk of damage accrual. Arthritis Rheumatol..

[35] Sammaritano L.R., Askanase A., Bermas B.L., Dall’Era M., Duarte-García A., Hiraki L.T., Son M.B.F., Werth V.P., Aranow C., Barnado A. (2026). 2025 American College of Rheumatology (ACR) Guideline for the Treatment of Systemic Lupus Erythematosus. Arthritis Rheumatol..

[36] Peschken C.A., Wang Y., Abrahamowicz M., Pope J., Silverman E., Hudson M., Arbillaga H., Smith C.D., Zummer M., Pineau C.A. (2019). Persistent Disease Activity Remains a Burden for Patients with Systemic Lupus Erythematosus. J. Rheumatol..

[37] Zen M., Iaccarino L., Gatto M., Saccon F., Larosa M., Ghirardello A., Doria A. (2017). The effect of different durations of remission on damage accrual: Results from a prospective monocentric cohort of Caucasian patients. Ann. Rheum. Dis..

[38] Mak A., Cheung M.W., Chiew H.J., Liu Y., Ho R.C. (2012). Global trend of survival and damage of systemic lupus erythematosus: Meta-analysis and meta-regression of observational studies from the 1950s to 2000s. Semin. Arthritis Rheum..

[39] Mok C.C., Ho L.Y., Chan K.L., To C.H. (2020). Trend of survival of a cohort of Chinese patients with systemic lupus erythematosus over 25 Years. Front. Med..

[40] Cervera R., Khamashta M.A., Font J., Sebastiani G.D., Gil A., Lavilla P., Mejía J.C., Aydintug A.O., Chwalinska-Sadowska H., de Ramón E. (2003). Morbidity and mortality in systemic lupus erythematosus during a 10-year period: A comparison of early and late manifestations in a cohort of 1000 patients. Medicine.

